# Facilitation of trace metal uptake in cells by inulin coating of metallic nanoparticles

**DOI:** 10.1098/rsos.170480

**Published:** 2017-09-13

**Authors:** Esmeralda Santillán-Urquiza, Fernando Arteaga-Cardona, Cristina Torres-Duarte, Bryan Cole, Bing Wu, Miguel A. Méndez-Rojas, Gary N. Cherr

**Affiliations:** 1 Departamento de Ingeniería Química, Ambiental y de Alimentos, Universidad de las Américas Puebla, Puebla, Mexico; 2 Departamento de Ciencias Químico-Biológicas, Universidad de las Américas Puebla, Puebla, Mexico; 3 Bodega Marine Laboratory, University of California-Davis, Bodega Bay, CA, USA; 4 School of Veterinary Medicine, University of California-Davis, Davis, CA, USA; 5 Departments of Environmental Toxicology and Nutrition, University of California-Davis, Davis, CA, USA; 6 School of the Environment, Nanjing University, Nanjing, People's Republic of China

**Keywords:** nanotoxicity, mussel haemocytes, zinc oxide nanoparticles, inulin coating, food fortification

## Abstract

Trace elements such as zinc and iron are essential for the proper function of biochemical processes, and their uptake and bioavailability are dependent on their chemical form. Supplementation of trace metals through nanostructured materials is a new field, but its application raises concerns regarding their toxicity. Here, we compared the intracellular zinc uptake of different sources of zinc: zinc sulfate, and ZnO and *core-shell* α-Fe_2_O_3_@ZnO nanoparticles, coated or uncoated with inulin, an edible and biocompatible polysaccharide. Using mussel haemocytes, a well-known model system to assess nanomaterial toxicity, we simultaneously assessed zinc accumulation and multiple cellular response endpoints. We found that intracellular zinc uptake was strongly enhanced by inulin coating, in comparison to the uncoated nanoparticles, while no significant effects on cell death, cell viability, mitochondrial membrane integrity, production of reactive oxygen species or lysosome abundance were observed at concentrations up to 20 ppm. Since no significant increments in toxicity were observed, the coated nanomaterials may be useful to increase *in vivo* zinc uptake for nutritional applications.

## 1. Introduction

Food deficiencies of trace elements (micronutrients) such as zinc, iron, calcium and copper are a cause of concern due to the implications that these may have on human health [1–3]. Fortification of foods is considered a convenient and general strategy to solve the deficiency of such nutrients. However, this application is limited by several problems such as the choice of the chemical form of the micronutrient, and the sensorial impact that it may have on taste or presentation, stability or bioavailability [4]. Biocompatible engineered nanomaterials (ENMs) are considered an option to overcome most of these problems [5,6]. The design and preparation of biocompatible, stable and easily dispersible inorganic nanoparticles for iron and zinc fortification is a promising and active field [7]. Polysaccharide chains such as inulin, a polydisperse polysaccharide that has been widely used as a stabilizer and excipient in the food and pharmaceutical industries, can be used as surfactants to prepare biocompatible, water soluble and stable nanoparticles [8]. Following this strategy, we have recently reported the synthesis and physical characterization of inulin coated and uncoated ZnO and *core-shell* α-Fe_2_O_3_@ZnO nanoparticles for food fortification as a source of biocompatible zinc and iron [2].

However, the use of nanomaterials raises concerns about their potential risks. Toxicity assessment of any new application of a chemical must be performed before it is used in commercial products, and this is particularly critical for ENMs, because any modifications of their properties, such as size, surface area, shape and crystallinity, among several others, can significantly impact their toxicity [9,10]. The use of cell cultures for *in vitro* testing of ENMs, toxicity has been extensively documented [11–17]. As an initial approach to determine the compatibility in cellular systems, we investigated the effects of inulin coated and uncoated ZnO and α-Fe_2_O_3_@ZnO nanoparticles in mussel haemocytes, the primary circulating cells of the mussel's blood (haemolymph) that have a similar structure and function to the mammalian macrophage [18,19]. This model system has proven to be an inexpensive and easy to handle model system to evaluate multiple cellular endpoints in response to exposure to ENMs [19–21]. Using a newly developed high-content screening system, we simultaneously assessed multiple endpoints including cell death, cell viability, mitochondrial membrane integrity, production of reactive oxygen species, lysosome abundance and zinc uptake. With this novel approach, we determined that no cellular responses were significantly affected, while zinc uptake was increased in nanoparticle-treated cells, particularly for inulin coated nanoparticles. The correlation between the physicochemical properties of the nanoparticles and mechanisms of intracellular zinc uptake is discussed.

## 2. Experimental section

### 2.1. Chemicals

Zinc oxide nanoparticles (nZnO) and haematite (α-Fe_2_O_3_) coated zinc oxide nanoparticles (nα-Fe_2_O_3_@ZnO), with or without inulin coating (smaller than 80 nm) were prepared following the procedure reported by Santillán-Urquiza *et al.* [2]. A detailed description of the preparation method and the characterization of these nanomaterials can be found in the electronic supplementary material, and the main properties are summarized in table 1. The fluorescent probes Hoechst 33342, Lysotracker Red DND-99, 5,5′,6,6′-tetrachloro-1,1′,3,3′-tetraethylbenzimidazolylcarbocyanineiodide (JC1), 2′,7'-dichlorodihydrofluorescein diacetate (DCF), calcein-AM (CAM), ethidium homodimer-1 (EtHD), Bioparticle Alexa Fluor 488 conjugated to *Saccharomyces cerevisae*, FluoZin-3 tetrapotassium salt and Newport Green (NPG) diacetate were purchased from Life Technologies (Carlsbad, CA) and stored in aliquots in a freezer at −20°C until their use. Paraformaldehyde was obtained from Electron Microscopy Sciences (Hatfield, PA). All other chemicals were purchased from Sigma-Aldrich (St Louis, MO) and were used as received.

**Table 1.** Properties of the ZnO and α-Fe2O3@ZnO nanoparticles and their inulin coated versions. TEM, transmission electron microscopy; PS-FBS1%, physiological saline supplemented with 1% fetal bovine serum.

	nZnO	nZnO inulin	nα-Fe_2_O_3_@ZnO	nα-Fe_2_O_3_@ZnO inulin
size (by TEM, nm)	70	92	50	14
zinc content (% w/w)^a^	77.2	73.8	47.8	43.3
iron content (% w/w)^a^	0	0	28.2	25.5
inulin content (% w/w)	0	8.1	0	9.5
hydrodynamic size ranges in DI (nm)	76–1150	100–246	70–300	60–150
hydrodynamic average size in DI (nm)	200	150	221	85
hydrodynamic size ranges in PS-FBS1% (nm)	1114–1800	818–1635	102–972	243–486
hydrodynamic average size in PS-FBS1% (nm)	1457	1071	449	324
zinc dissolution in PS after 15 min (%)^b^	23	34	13	24.5
zinc dissolution in PS after 120 min (%)^b^	22	35.5	14	30

^a^From energy dispersive spectroscopy (EDS) data (average of several area determinations) analysis.

^b^From a dispersion containing a total of 20 ppm of Zn. pH was 7.3.

### 2.2. Animals and haemocytes collection

Adult mussels (*Mytillus galloprovincialis*, length 7.1 ± 0.97 cm) were purchased from the Bodega Bay Oyster Company (Bodega Bay, CA) and were maintained at the University of California Davis Bodega Marine Laboratory (Bodega Bay, CA) in flow-through seawater tanks for two days before haemolymph extraction. In order to extract the haemocytes non-lethally, a notch was formed in the mussel shell with a triangular file and 0.5 ml of haemolymph was aspirated from the posterior adductor muscle using a sterile syringe containing 0.5 ml of ice-cold physiological saline (PS) Ca/Mg-free (20 mM HEPES, 436 mM NaCl, 10 mM KCl, 53 mM Na_2_SO_4_ and 0.5 mM EDTA, pH value was adjusted to 7.3 using NaOH). Each aspirated haemolymph was examined with an optical microscope for the presence of haemocytes, determined cell density using a haemocytometer and maintained on ice to avoid clumping. After haemolymph extraction from 3–10 mussels, haemocytes were pooled and density was adjusted to a stock concentration of 5 × 10^5^ cells ml^−1^ by diluting the cells with PS Ca/Mg-free. Hoechst 33342 (1 µM final concentration) was added to stain the cell nucleus. To prepare haemocytes monolayers, 100 µl of haemocytes stock was added into the wells of 96-well plates previously equilibrated at 14°C with 150 µl of PS (20 mM HEPES, 436 mM NaCl, 10 mM KCl, 53 mM MgSO_4_, 10 mM CaCl_2_, pH adjusted to 7.3 using NaOH). Plates were incubated for 45 min at 14°C to allow cell attachment and spreading.

### 2.3. *In vitro* chemical exposure of mussel haemocytes

Stock solutions (1 g l^−1^) of different zinc sources: nZnO, nα-Fe_2_O_3_@ZnO, with and without inulin coating, ZnSO_4_ (as a positive control of a soluble salt) and pure inulin, were prepared in deionized (DI) water and then sonicated in a Branson model 2510 sonic bath (Danbury, CT) for 30 min (max. 100 W). For the nanoparticle treatments, the concentration was normalized considering only the zinc percentage in each sample, so all the solutions would have the same zinc concentration regardless of their composition. The zinc percentage in each sample was obtained by the results of atomic absorption spectroscopy (see electronic supplementary material). The unfiltered stock solutions were then diluted into PS supplemented with 1% fetal bovine serum (FBS) to disperse the chemicals (nanoparticles, zinc sulfate or inulin). Six concentrations of each system (0, 1, 2.5, 5, 10 and 20 ppm) were prepared. To analyse the cellular responses due to chemical exposure, 200 µl per well of each target system was added to the adherent haemocytes monolayer (two columns per concentration). Exposures lasted for 120 min at 14°C with gentle mixing.

### 2.4. Use of fluorescent probes for toxicity evaluations

Different cellular responses were analysed to determine the potential toxic effects of the chemicals using intracellular fluorescent probes. Their properties and targets are listed in table 2.

**Table 2.** Fluorescent probes used to analyse different cellular responses.

probe	final concentration	excitation	emission	parameter analysed
Hoechst 33342	0.2 µM	360 nm	465 nm	permeable nuclear stain for total cell number
ethidium homodimer-1 (EtHD)	10 µM	530 nm	630 nm	impermeable nuclear stain for cell death: staining of nuclei of cells only when plasma membrane is damaged
JC1	1 µg ml^−1^	485 nm	535 nm	mitochondria membrane potential
Lysotracker Red DND-99	0.1 µM	560 nm	630 nm	lysosome abundance
2′,7′-dichlorofluorescin diacetate (DCF)	10 µM	485 nm	535 nm	production of intracellular reactive oxygen species
calcein-AM (CAM)	2.5 µM	485 nm	535 nm	assesses intracellular esterase activity through production of fluorescent calcein
Newport Green (NPG)	0.5 µM	485 nm	535 nm	soluble intracellular zinc concentration

The probes were added to the cells in the wells of plates in the presence of the corresponding chemical treatments (two rows per probe). Lysotracker was added at the same time as the chemical treatment. After 75 min of incubation, JC1, DCF, CAM and EtHD were added; CAM and EtHD were added to the same rows. Then, 25 min later, NPG was added to the rows containing Lysotracker. After incubation for an additional 20 min, the plates were washed three times with 200 µl per well of PS. The fluorescence was detected using a Tecan GENios microplate reader (Maennedorf, Switzerland) at the emission and excitation wavelengths indicated in table 2. Each combination of target chemical concentration and probe was internally replicated in four wells of each plate, and each exposure plate was replicated four times using a different batch of pooled haemocytes collected from different mussels.

### 2.5. Phagocytosis assays

To analyse cell function in the presence of different sources of zinc, haemocyte monolayers (200 000 haemocytes per well) were prepared on a 24-well plate, as described in §2.2. After elimination of non-adherent haemocytes, 1 ml of the corresponding treatment solution was added to a final concentration of 20 ppm along with Alexa-488 yeast (10 yeast/haemocyte). Inulin and PS without nanoparticles were used as controls. Each treatment was replicated in three wells. After incubation at 14°C for 15 min, excess yeast was washed twice with PS and samples were fixed with paraformaldehyde (0.1% v/v final concentration) before imaging using fluorescence (excitation at 488 nm) in a Nikon AZ100 macrozoom stereo fluorescence microscope (Melville, USA) at 20× magnification. Images were saved and later visually analysed to determine the percentage of haemocytes (*n* > 50 haemocytes per well) that contained at least one fluorescently labelled yeast inside of the cell (phagocytized).

### 2.6. Analysis of zinc accumulation in tissue

To determine the total amount of zinc accumulated inside the haemocytes, haemocyte monolayers (800 000 haemocytes per well) were prepared on a six-well plate, as described in §2.2. After elimination of non-adherent haemocytes, 10 ml of the corresponding treatment solution with a concentration of 20 ppm was added. Inulin and PS without nanoparticles were used as controls. After incubation for 2 h at 14°C, plates were washed four times with PS to eliminate any residual treatment solution. Wells were visually inspected using an inverted microscope throughout the process to determine adherence of haemocytes, with no detectable differences due to either washes or treatment with the corresponding chemical. Tissue was homogenized in 1.9 ml of metal-free filtered seawater pH 1.6 (acidified with HCl trace metal grade) by sonication directly in the well with a probe for at least 2 min, until no intact cells were observed under the microscope. The homogenized tissue was transferred to a 2 ml microcentrifuge tube and samples were incubated for 4 h at room temperature to allow the metal ions to dissolve. Then, samples were centrifuged at 10 000*g* for 10 min to eliminate cell debris. Supernatant was transferred to a fresh tube and stored at 4°C until analysis. Each target chemical was internally replicated in two wells of each plate, and measurements were replicated two times using a different batch of pooled haemocytes collected from different mussels.

Zinc concentration in cell extracts was determined by fluorescence using a modified version of the method reported by Grand *et al*. [22] which was used for the determination of zinc in seawater and has been adapted for a plate reader. Details are provided in the electronic supplementary material, figure S1. Zinc concentrations in each sample were normalized by the number of cells in each well.

### 2.7. Confocal microscopy

For imaging, haemocyte monolayers were prepared in a chamber slide system (1.7 cm^2^ per well, 200 000 haemocytes per well). After elimination of non-adherent haemocytes, 800 µl of the corresponding treatment solution with a concentration of 20 ppm was added. The addition of the corresponding fluorescent probe was done following the same conditions described for the plate reader assay (§2.4). After incubation for 2 h at 14°C, slides were washed four times with PS to eliminate any residual treatment solution. Before imaging, haemocytes were fixed with paraformaldehyde (2% final concentration). After incubation for 10 min at room temperature, the liquid was eliminated and the slides were separated and compressed under cover glass, then viewed with a 20× water immersion lens on an Olympus BX61WI fixed stage upright microscope using scanning laser confocal microscopy using both interference contrast optics (transmitted light, DIC) and the corresponding excitation wavelengths as described previously [23].

### 2.8. Data analysis

For analysis of cellular responses using the plate reader assay, fluorescence intensity values obtained with the different cellular probes were first normalized by cell number in each well by dividing the fluorescence intensity of the probe by the fluorescence intensity values of Hoechst 33342. The normalized fluorescence value of each well was averaged with the results from the same conditions (four wells per concentration and probe combination). Then, the average was divided by the response from the control treatment (no chemical exposure) to calculate the relative probe response between target and control group.

Results were statistically evaluated by one-way analysis of variance test. For results from the cellular responses measured in the plate reader, Dunnett's post hoc test was performed for comparisons to control treatments. For internalized zinc concentration and phagocytic activity, Tukey's post hoc test was performed for multiple comparisons. Statistical significance was set at *α* = 0.05. Analyses were performed using GraphPad Prism 6.0 (GraphPad Software, Inc., La Jolla, CA).

## 3. Results and discussion

### 3.1. Mussel haemocytes as a model for ENMs toxicity evaluation

There are numerous commercially available fluorescent dyes that can be used to analyse biological cellular responses. EthD-1 is used to determine cell death as it can only enter cells with damaged membranes and its fluorescence increases 40-fold when bound to nucleic acids. Cell viability was determined with CAM, a non-fluorescent membrane permeable dye which upon entering the cell is cleaved by intracellular esterases releasing the non-permeable and strongly fluorescent calcein. JC-1 is used to monitor mitochondrial health as it indicates changes in the membrane potential by changing fluorescence from red to green. Lysotracker Red is a fluorescent acidotropic probe used to detect the lysosomes, which are acidic organelles. H2DCFDA is a fluorescent probe employed to detect reactive oxygen species (ROS), including hydrogen peroxide, hydroxyl radicals and peroxynitrile. Upon oxidation by ROS, the non-fluorescent H2DCFDA is converted to the highly fluorescent DCF. NPG is a cell-permeant probe that selectively binds to Zn(II) and was used to investigate the accumulation of intracellular Zn(II). The use of these fluorescent probes to determine cellular responses *in vitro* has been previously reported for toxicity analysis [24–27].

Here, we adopted the use of these fluorescent probes to develop a high-content screening (multiple endpoints assessed simultaneously) to determine the impacts of ENMs in mussel haemocytes, cells that can be used as a model system to study the effects of ENMs in marine organisms as well as the cellular toxicity responses [18,20]. The approach used here is similar to that used in previous studies on mammalian cell lines [11]. To validate this new methodological approach, first we analysed the effects of silver and copper oxide nanoparticles, as well as single-walled carbon nanotubes, materials that have been previously demonstrated to be toxic using other model systems (see electronic supplementary material) [23,28–30]. Results show that exposure of mussel haemocytes to toxic ENMs can significantly affect cell parameters such as phagocytosis, lysosome abundance, production of reactive oxygen species and cell viability (electronic supplementary material, figure S2). Haemocytes are viable for up to 24 h when cultured under ideal conditions; however, these results show that a 2 h exposure is enough to detect cellular damage caused by toxic nanoparticles, further demonstrating that this is a useful model system to evaluate multiple cellular toxicity parameters using a high-content screening method.

### 3.2. Cellular responses to nZnO and nα-Fe_2_O_3_@ZnO exposure

Coating of a nanoparticle surface is a general strategy used to increase stability, biocompatibility and water solubility as well as to decrease immunogenic response or toxicity. Several inorganic and organic coating materials have been explored to achieve that, including silica (SiO_2_), gold (Au), titania (TiO_2_), zirconia (ZrO_2_), dextran and polyethylene glycol among several others [31]. Here, we evaluated the effect of inulin coating on two different nanoparticles, nZnO and nα-Fe_2_O_3_@ZnO [2]. Nanoparticles coated with inulin showed better dispersion than those uncoated, both in water and in PS supplemented with 1% fetal bovine serum (PS-FBS1%) (table 1). In DI water, nZnO has an average size of 200 nm (76–1150 nm) while nZnO inulin has an average of 150 nm (100–246 nm). When suspended in PS-FBS1%, nZnO has an average size of 1457 nm (1114–1800 nm), while nZnO inulin has an average of 1071 nm (818–1635 nm). For nα-Fe_2_O_3_@ZnO the average size in DI water was 221 nm (70–300 nm) and 85 nm (60–150 nm) for the uncoated and inulin coated version, respectively, while in PS-FBS1%, inulin uncoated and coated nα-Fe_2_O_3_@ZnO nanoparticles were 449 nm (102–972 nm) and 324 nm (243–486 nm), respectively. As it was expected, when dispersed in PS-FBS1%, nanoparticle aggregates had a larger size distribution due to increased ionic strength and the presence of proteins, an effect that has been documented in different cell culture media [32]. However, inulin coating partially allowed a better dispersion of the nanoparticles by preventing the formation of large aggregates, maintaining a narrower size distribution as well as a smaller average size.

**Figure 1.**
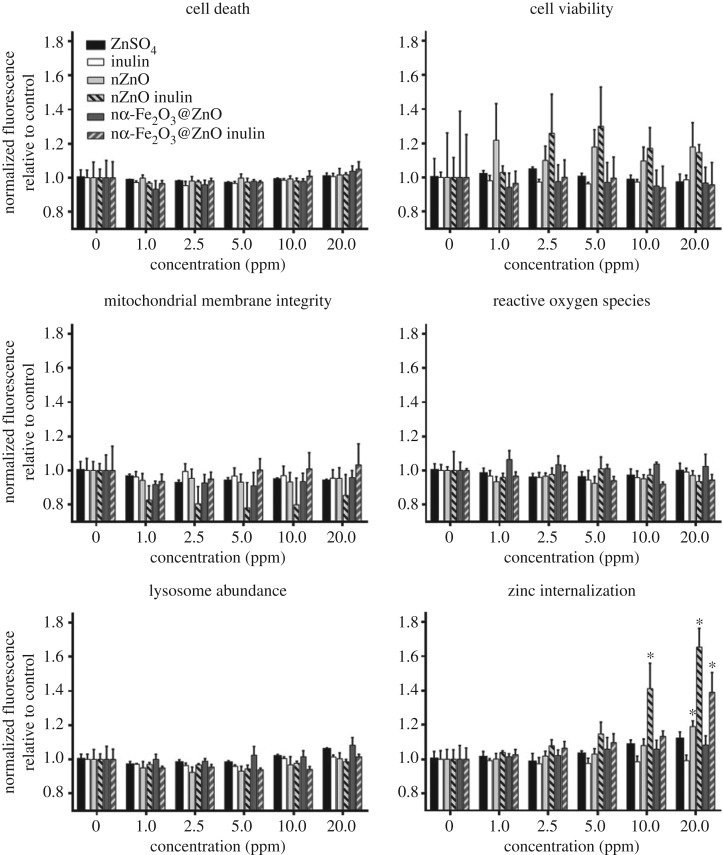
Cellular responses of mussel haemocytes exposed to ZnO and α-Fe_2_O_3_@ZnO and their inulin coated versions. No statistically significant changes were induced on cell death, cell viability, mitochondrial membrane integrity, production of reactive oxygen species or lysosome abundance (*p* > 0.05). nZnO inulin causes the highest increase in zinc internalization measured an increased NPG fluorescence, starting at 10 ppm. Treatment with 20 ppm of nZnO or nα-Fe_2_O_3_@ZnO inulin also increase NPG fluorescence. The asterisk indicates statistically significant differences (*p* < 0.05) compared with the 0 ppm (control) treatment of the corresponding chemical treatment according to Dunnett's comparison. Values are average ± s.e. (*n* ≥ 4).

In this study, no adverse responses by haemocytes were detected in cells exposed to nZnO and nα-Fe_2_O_3_@ZnO or their inulin coated versions at concentrations up to 20 ppm (figure 1) for periods of two hours. The endpoints analysed included cell death, cell viability, mitochondrial membrane integrity, ROS production and lysosomal abundance. The only parameter (non-toxicological) that was significantly affected with nanoparticle treatment was zinc internalization (figure 1). Fluorescence of NPG, a fluorescent probe selective to intracellular soluble zinc, slightly increased in haemocytes exposed to 20 ppm of nZnO, while inulin coating of nZnO had a significant effect when present at 10 and 20 ppm. Also, treatment with nα-Fe_2_O_3_@ZnO had no significant effect on NPG fluorescence, but coating with inulin did have a significant effect when present at 20 ppm. This indicates that inulin coating has an effect on intracellular soluble zinc concentration in the haemocytes without affecting other important cellular functions.

### 3.3. Inulin coating of nZnO and nα-Fe_2_O_3_@ZnO increases zinc uptake

**Figure 2.**
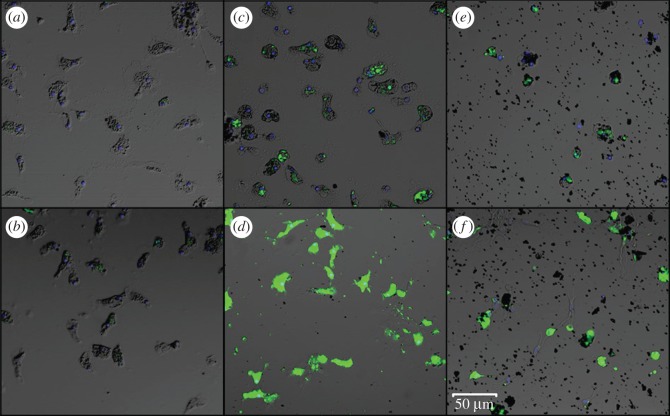
Confocal images of the haemocytes (*a*) control, and treated with a 20 ppm of (*b*) ZnSO_4_, (*c*) nZnO, (*d*) nZnO inulin, (*e*) nα-Fe_2_O_3_@ZnO and (*f*) nα-Fe_2_O_3_@ZnO inulin. An increase in green fluorescence indicates a higher concentration of soluble zinc inside the haemocytes detected with NPG (0.5 µM). Hoechst 33342 (blue) was used to detect the cell nucleus of each individual haemocyte. Scale bar, 50 µm.

More direct evidence that inulin coated ENM samples increase the amount of intracellular soluble zinc was observed using confocal microscopy of haemocytes exposed to the highest concentration (20 ppm) of the corresponding treatments. As can be seen in figure 2*a*, no fluorescence of NPG (shown in green) is observed in untreated haemocytes, while treatment with ZnSO_4_ as a source of soluble zinc caused a very slight increase in fluorescence of NPG in some haemocytes, observed as dim green dots (figure 2*b*). When haemocytes were treated with nZnO (figure 2*c*) and α-Fe_2_O_3_@ZnO (figure 2*e*), the increase in green fluorescence was more visible, while treatments with nZnO inulin (figure 2*d*) and nα-Fe_2_O_3_@ZnO inulin (figure 2*f*), showed the highest fluorescence of all. The results from confocal microscopy strongly correlate with the increase in fluorescence measured with the plate reader.

**Figure 3.**
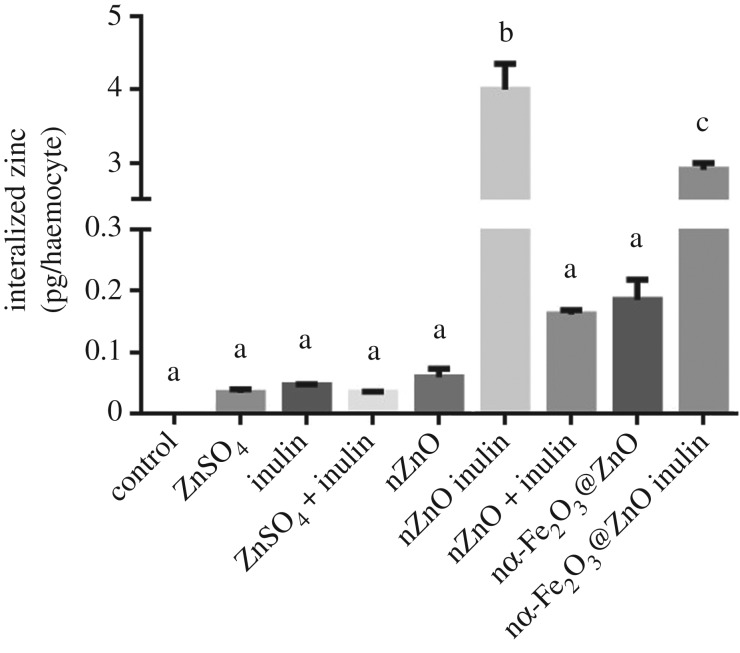
Total zinc concentration measured by fluorescence in haemocytes exposed for 2 h to 20 ppm of the corresponding chemical. Inulin coating of nZnO and nα-Fe_2_O_3_@ZnO cause a significant increase in zinc internalization. Different lower case letters represent statistical differences between treatments (*p* < 0.05) according to Tukey test. Values are average ± s.e.

NPG is a fluorescent probe that selectively binds to intracellular soluble Zn(II), and thus is not able to detect zinc in a nanoparticulate form, as has been previously documented in mammalian cells exposed to zinc oxide nanoparticles [26]. For haemocytes, it is very likely that there are nanoparticle aggregates that have been internalized through phagocytosis and that had not been detected through NPG fluorescence. To determine the total amount of zinc internalized by the haemocytes, whether in soluble or nanoparticulate form, we homogenized the treated cells and acidified them to solubilize all the possible remaining nanoparticles that would have been internalized. As can be seen in figure 3, large amounts of zinc can be found for both inulin coated nZnO and nα-Fe_2_O_3_@ZnO, in comparison to the uncoated systems or even in haemocytes exposed to the highly soluble zinc sulfate. Inulin coating of nZnO caused a 67-fold increase in zinc accumulation as compared to the uncoated nanoparticle, while haemocytes treated with nα-Fe_2_O_3_@ZnO inulin accumulated 15 times more zinc than haemocytes treated with nα-Fe_2_O_3_@ZnO. All results suggest that zinc internalization increases were more significant for samples coated with inulin than the uncoated ones, and cannot be attributed solely to the presence of inulin in the media, as there were no significant differences in zinc concentrations in haemocytes treated with nZnO in the presence of soluble inulin in the media. This increase suggests that inulin coating plays an important role in nZnO uptake, either by increasing the dissolution of the nanoparticles, the bioavailability of zinc, or by increasing the active internalization of the nanoparticles by phagocytosis.

### 3.4. Exploring mechanisms for increased zinc uptake

Zinc oxide nanoparticles have been previously demonstrated to dissolve easily in aqueous media [33,34]. However, many characteristics of the nanoparticles determine their behaviour, including size, shape, surface area and functionalization [9]. To determine if inulin coating plays a role in nanoparticle dissolution, we measured the amount of soluble zinc present in the media once dispersed in PS. After 2 h, 22% of nZnO was dissolved while for nZnO inulin 35% of zinc ions dissolved. Similar results were obtained for nα-Fe_2_O_3_@ZnO and nα-Fe_2_O_3_@ZnO inulin, where 14% and 30% dissolution were obtained, respectively (table 1). This indicates that inulin coating does increase nanoparticle dissolution, causing them to release higher amounts of soluble zinc in the media. However, this cannot solely explain the dramatic increase in zinc uptake observed in figure 3 as haemocytes treated with ZnSO_4_ were the ones exposed to the highest concentration of soluble zinc (20 ppm) and accumulated less zinc than haemocytes treated with inulin coated nanoparticles (7.1 ppm of soluble zinc for nZnO inulin and 6.0 ppm of soluble zinc for nα-Fe_2_O_3_@ZnO inulin).

**Figure 4.**
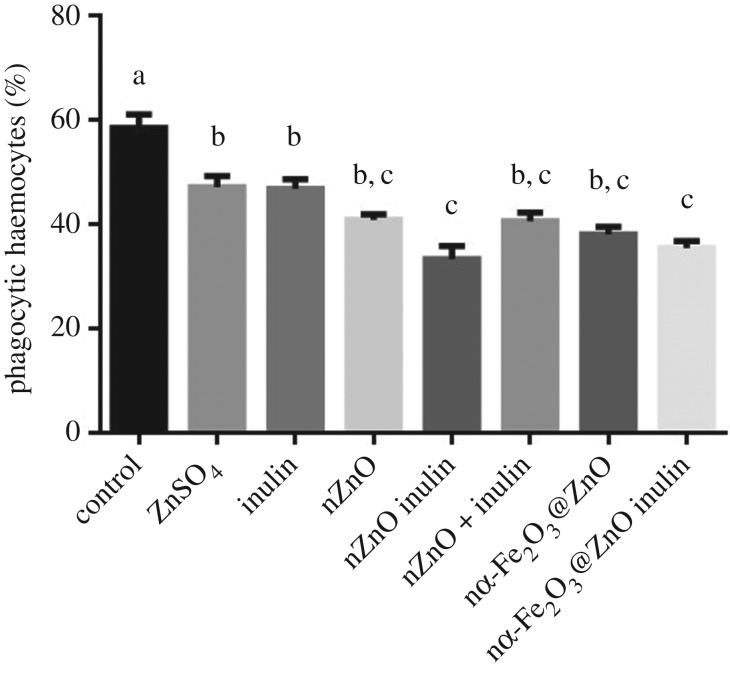
Percentage of phagocytic haemocytes after 15 min of exposure to 20 ppm of either zinc sulfate, inulin or nanoparticles. Different lower case letters represent statistical differences between treatments (*p* < 0.05) according to Tukey test. Values are average ± s.e.

We hypothesized that active nanoparticle uptake would be the primary cause of increased zinc uptake. The biological role of haemocytes is to defend the mussel from pathogens and parasites, mainly by elimination through phagocytosis [18]. Size plays an important role in detection of foreign objects, with haemocytes preferentially engulfing particles of at least 1 µm, which is the average size of the nZnO and nZnO inulin aggregates formed in PS-FBS1% (table 1). Nanoparticle aggregates of this size can potentially stimulate phagocytosis thus increasing zinc uptake. To test this, phagocytosis was analysed in the presence of 20 ppm of the corresponding nanoparticle. To test if soluble inulin stimulates phagocytosis, an extra control was used by simultaneously adding uncoated nZnO with free inulin. It was observed that the presence of either zinc sulfate, inulin or any of the nanoparticles tested, decreased phagocytosis (figure 4), and contrary to what was expected, it appeared that inulin coating further decreased phagocytic activity. This effect cannot be attributed to the presence of uncoated nZnO and uncoated nZnO plus free inulin, which showed similar phagocytic activity.

From figure 4 it can be inferred that the pathway for increasing zinc internalization is not based on stimulation of total phagocytic activity. However, it is possible that haemocytes are preferentially phagocytosing inulin coated nanoparticles while ignoring the fluorescently labelled yeast. It has been demonstrated that the chemical composition of the bacterial cell surface plays an important role in recognition and ingestion by haemocytes [18], so it is possible that inulin coating increases the selectivity towards the nanoparticles. Furthermore, the presence of FBS as a supplement to increase the nanoparticle stability in solution can contribute to the modification of the nanoparticle surface, as FBS contains numerous biomolecules (sugars, lipids, proteins, among others). The interaction of nanoparticles and biomolecules (in particular proteins) results in the formation of a biological corona on the nanoparticle's surface; these coronas are usually long-lived and may affect how nanoparticles and cells interact, as reported recently [35–38]. Also, some proteins in the corona may target specific receptors on the cell membrane, activating biological responses such as inflammation, toxicity, bioavailability, nanoparticle uptake, among others, which do not happen by the presence of the nanoparticle and/or surfactant alone [39]. Once the particles enter a cell, removal of the protein corona and the inulin coating can be expected as a consequence of lysosome action [36], increasing the bioavailability of the nanoparticle chemical components.

The results obtained by NPG fluorescence as well as the dissolution values in PS indicate that inulin coating has an effect in dissolution, whether in the extracellular media or inside the cells. To further test this, dissolution was analysed in deionized water to determine the direct effect of inulin and eliminate any possible interference of the many components present in the exposure media, PS-FBS 1%. Analysis were conducted at different pHs that mimic different scenarios: neutral conditions present in the extracellular media (pH 7), pH 4.5 that simulates the pH found in lysosomes, and pH 2 that simulates the acidic conditions of human digestive tract.

**Figure 5.**
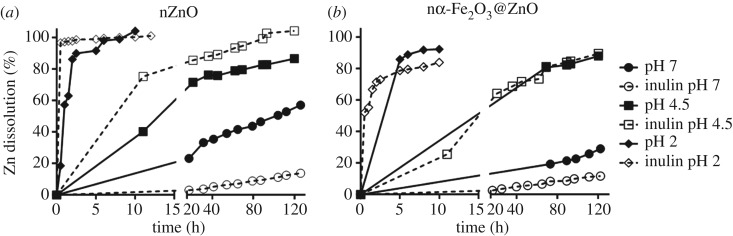
Release of Zn(II) ions in deionized water at pH 7 (circle), pH 4.5 (square) and pH 2 (diamond) for uncoated (solid line) and inulin coated (open symbols, dashed lines) ZnO (*a*) and nα-Fe_2_O_3_@ZnO (*b*). At pH 7, inulin coating increases stability of both nanoparticles. At lower pHs, inulin coating of nZnO increases dissolution, while for nα-Fe_2_O_3_@ZnO there are no significant changes.

Figure 5 shows that at any given condition, nZnO dissolves faster than nα-Fe_2_O_3_@ZnO. This is in accordance with results obtained in other studies where iron doping of nZnO decreased nanoparticle dissolution and toxicity [40]. Also, at neutral pH, inulin coating decreased dissolution demonstrating the protective effect of inulin. As expected, release of Zn(II) ions increases as pH decreases. At pH 2 and 4.5, half of the nanoparticles dissolved into Zn(II) within the first 24 h. Interestingly, inulin coating seems to have contrasting effects in dissolution at low pHs. For nZnO, inulin coating further increased dissolution, while for nα-Fe_2_O_3_@ZnO inulin coating had no effect at pH 4.5 and decreased dissolution at pH 2. Glibowski and Bukowska [41] reported that inulin degrades at acidic pH, and given that inulin is at the surface of the nanoparticles, its degradation would destabilize the nanoparticles, potentially increasing their dissolution. This would be critical for nZnO because it is initially more soluble when uncoated, but inulin dissolution would not significantly increase the release of Zn(II) ions for nα-Fe_2_O_3_@ZnO because its dissolution is lower due to the complexation with haematite. Also, the degradation of inulin itself can affect dissolution by altering the properties of the media by releasing fructose monomers and oligomers, competing with the releasing of free Zn(II) ions from ZnO by formation of stable metal complexes among Zn(II) and the degradation products. It has been previously demonstrated that the presence of organic material in the media affects the dissolution of ENMs, an effect that is also pH dependent [42].

As inulin coated nanoparticles are more stable in the media than uncoated ones, they are also more bioavailable, which can lead to increased endocytosis [43], and can become more prone to recognition of the nanoparticle surface and preferential phagocytosis by the haemocytes over other particles present in the media [18]. Once inside the haemocytes, inulin coated nanoparticles would dissolve much faster in lysosomes where the lower pH might accelerate Zn(II) released from the inorganic nanoparticles. In summary, this behaviour suggests that nZnO inulin, after internalization in the haemocytes, are able to release more zinc ions than the uncoated materials. The faster dissolution rate for the inulin coated samples, compared with the uncoated samples, makes Zn(II) ions more available for the cells, which may be useful for different applications like food fortification, or reduce toxicity of nanomaterials.

## 4. Conclusion

A convenient, easy to use and reproducible biological model for the evaluation of biological impact of nanomaterials was used to analyse the cellular responses and toxicity of a set of inulin coated and uncoated inorganic nanoparticles (nZnO and nα-Fe_2_O_3_@ZnO). Using a novel approach, multiple important cell toxicity parameters (cell death, cell viability, mitochondrial membrane integrity, reactive oxygen species generation, lysosome abundance and metal uptake) were simultaneously evaluated.

Zinc uptake in cells was significantly increased by nanoparticle exposure while having no significant effects on the cellular toxicity endpoints analysed. Inulin coated nanoparticles further increased zinc uptake. These results suggest that inulin coated nZnO and nα-Fe_2_O_3_@ZnO may be good candidates for nutritional uses, such as the preparation of fortified foods. Further work aimed to evaluate the uptake of different metal ions using inulin as a coating material is currently being done.

## Supplementary Material

Nanoparticle preparation, zinc uptake assessment, toxicity determination

## Data accessibility

Complementary information supporting this article has been uploaded as part of the electronic supplementary material.

## References

[1] Yada RY et al. 2014 Engineered nanoscale food ingredients: evaluation of current knowledge on material characteristics relevant to uptake from the gastrointestinal tract. Compr. Rev. Food Sci. Food Saf. 13, 730–744. (doi:10.1111/1541-4337.12076) 10.1111/1541-4337.12076 33412698

[2] Santillán-Urquiza E, Arteaga-Cardona F, Hernandez-Herman E, Pacheco-García P, González-Rodríguez R, Coffer JL, Mendoza-Alvarez M, Vélez-Ruiz JF, Méndez-Rojas MA 2015 Inulin as a novel biocompatible coating: evaluation of surface affinities toward CaHPO_4_, α-Fe_2_O_3_, ZnO, CaHPO_4_ @ZnO and α-Fe_2_O_3_ @ZnO nanoparticles. J. Colloid Interface Sci. 460, 339–348. (doi:10.1016/j.jcis.2015.08.057) 26364076 10.1016/j.jcis.2015.08.057

[3] Santillan-Urquiza E, Ruiz-Espinosa H, Angulo-Molina A, Velez-Ruiz JF, Méndez-Rojas MA 2017 Applications of nanomaterials in functional fortified dairy products: benefits and implications for human health. In Nutrient Delivery, 1st edn (ed. Grumezescu A), pp. 293–328. London, UK: Academic Press.

[4] Domínguez R, Barreiro T, Sousa E, Bermejo A, Cocho JA, Fraga JM, Bermejo P 2004 Study of the effect of different iron salts used to fortify infant formulas on the bioavailability of trace elements using ICP-OES. Int. Dairy J. 14, 1081–1087. (doi:10.1016/j.idairyj.2004.03.011)

[5] Ravichandran R 2010 Nanotechnology applications in food and food processing: innovative green approaches, opportunities and uncertainties for global market. Int. J. Green Nanotechnol. Phys. Chem. 1, 72–96. (doi:10.1080/19430871003684440)

[6] Martirosyan A, Schneider YJ 2014 Engineered nanomaterials in food: implications for food safety and consumer health. Int. J. Environ. Res. Public Health 11, 5720–5750. (doi:10.3390/ijerph110605720) 24879486 10.3390/ijerph110605720 PMC4078545

[7] Hilty FM et al. 2010 Iron from nanocompounds containing iron and zinc is highly bioavailable in rats without tissue accumulation. Nat. Nanotechnol. 5, 374–380. (doi:10.1038/nnano.2010.79) 20418865 10.1038/nnano.2010.79

[8] Barclay T, Ginic-Markovic M, Cooper P, Petrovsky N 2010 Inulin: a versatile polysaccharide with multiple pharmaceutical and food chemical uses. J. Excipients and Food Chem. 1, 27–50.

[9] Nel AE, Mädler L, Velegol D, Xia T, Hoek EMV, Somasundaran P, Klaessig F, Castranova V, Thompson M 2009 Understanding biophysicochemical interactions at the nano–bio interface. Nat. Mat. 8, 543–557. (doi:10.1038/nmat2442) 10.1038/nmat2442 19525947

[10] Oberdörster G 2010 Safety assessment for nanotechnology and nanomedicine: concepts of nanotoxicology. J. Intern. Med. 267, 89–105. (doi:10.1111/j.1365-2796.2009.02187.x) 20059646 10.1111/j.1365-2796.2009.02187.x

[11] George S et al. 2010 Use of a rapid cytotoxicity screening approach to engineer a safer zinc oxide nanoparticle through iron doping. ACS Nano 4, 15–29. (doi:10.1021/nn901503q) 20043640 10.1021/nn901503q PMC3900637

[12] Gilbert B, Fakra SC, Xia T, Pokhrel S, Mädler L, Nel A 2012 The fate of ZnO nanoparticles administered to human bronchial epithelial cells. ACS Nano 6, 4921–4930. (doi:10.1021/nn300425a) 22646753 10.1021/nn300425a PMC4120753

[13] Hillegass JM, Shukla A, Lathrop SA, MacPherson MB, Fukagawa NK, Mossman BT 2010 Assessing nanotoxicity in cells *in vitro*. Wiley Interdiscip. Rev. Nanomed. Nanobiotechnol. 2, 219–231. (doi:10.1002/wnan.54) 20063369 10.1002/wnan.54 PMC2854858

[14] Joris F, Manshian BB, Peynshaert K, De Smedt SC, Braeckmans K, Soenen SJ 2013 Assessing nanoparticle toxicity in cell-based assays: influence of cell culture parameters and optimized models for bridging the *in vitro*–*in vivo* gap. Chem. Soc. Rev. 42, 8339–8359. (doi:10.1039/c3cs60145e) 23877583 10.1039/c3cs60145e

[15] Lanone S, Rogerieux F, Geys J, Dupont A, Maillot-Marechal E, Boczkowski J, Lacroix G, Hoet P 2009 Comparative toxicity of 24 manufactured nanoparticles in human alveolar epithelial and macrophage cell lines. Part. Fibre Toxicol. 6, 14 (doi:10.1186/1743-8977-6-14) 19405955 10.1186/1743-8977-6-14 PMC2685765

[16] Love SA, Maurer-Jones MA, Thompson JW, Lin YS, Haynes CL 2012 Assessing nanoparticle toxicity. Annu. Rev. Anal. Chem. 5, 181–205. (doi:10.1146/annurev-anchem-062011-143134) 10.1146/annurev-anchem-062011-143134 22524221

[17] Nirmal Suma R, Valappil Mohanan P 2015 Stem cells, a new generation model for predictive nano toxicological assessment. Curr. Drug. Metab. 16, 932–939. (doi:10.2174/1389200216666151015113720) 26467070 10.2174/1389200216666151015113720

[18] Canesi L, Gallo G, Gavioli M, Pruzzo C 2002 Bacteria-hemocyte interactions and phagocytosis in marine bivalves. Microsc. Res. Tech. 57, 469–476. (doi:10.1002/jemt.10100) 12112429 10.1002/jemt.10100

[19] Canesi L, Ciacci C, Fabbri R, Marcomini A, Pojana G, Gallo G 2012 Bivalve molluscs as a unique target group for nanoparticle toxicity. Mar. Environ. Res. 76, 16–21. (doi:10.1016/j.marenvres.2011.06.005) 21767873 10.1016/j.marenvres.2011.06.005

[20] Canesi L, Ciacci C, Vallotto D, Gallo G, Marcomini A, Pojana G 2010 *In vitro* effects of suspensions of selected nanoparticles (C60 fullerene, TiO_2_, SiO_2_) on *Mytilus* hemocytes. Aquat. Toxicol. 96, 151–158. (doi:10.1016/j.aquatox.2009.10.017) 19900724 10.1016/j.aquatox.2009.10.017

[21] Katsumiti A, Arostegui I, Oron M, Gilliland D, Valsami-Jones E, Cajaraville MP 2016 Cytotoxicity of Au, ZnO and SiO_2_ NPs using *in vitro* assays with mussel hemocytes and gill cells: relevance of size, shape and additives. Nanotoxicology 10, 185–193. (doi:10.3109/17435390.2015.1039092) 25962683 10.3109/17435390.2015.1039092

[22] Grand M, Oliveira HM, Ruzicka J, Measures C 2011 Determination of dissolved zinc in seawater using micro-Sequential Injection lab-on-valve with fluorescence detection. Analyst 136, 2747–2755. (doi:10.1039/c1an15033b) 21589977 10.1039/c1an15033b

[23] Wu B, Torres-Duarte C, Cole B, Cherr GN 2015 Copper oxide and zinc oxide nanomaterials act as inhibitors of multidrug resistance transport in sea urchin embryos: their role as chemosensitizers. Environ. Sci. Technol. 49, 5760–5770. (doi:10.1021/acs.est.5b00345) 25851746 10.1021/acs.est.5b00345

[24] Wang Z, Li N, Zhao J, White JC, Qu P, Xing B 2012 CuO nanoparticle interaction with human epithelial cells: cellular uptake, location, export, and genotoxicity. Chem. Res. Toxicol. 25, 1512–1521. (doi:10.1021/tx3002093) 22686560 10.1021/tx3002093

[25] Weston SA, Parish CR 1990 New fluorescent dyes for lymphocyte migration studies. J. Immunol. Methods 133, 87–97. (doi:10.1016/0022-1759(90)90322-M) 2212694 10.1016/0022-1759(90)90322-m

[26] Xia T, Kovochich M, Liong M, Mädler L, Gilbert B, Shi H, Yeh JI, Zink JI, Nel AE 2008 Comparison of the mechanism of toxicity of zinc oxide and cerium oxide nanoparticles based on dissolution and oxidative stress properties. ACS Nano 2, 2121–2134. (doi:10.1021/nn800511k) 19206459 10.1021/nn800511k PMC3959800

[27] Zhang H et al. 2014 PdO doping tunes band-gap energy levels as well as oxidative stress responses to a CO_3_O_4_ *p* -type semiconductor in cells and the lung. J. Am. Chem. Soc. 136, 6406–6420. (doi:10.1021/ja501699e) 24673286 10.1021/ja501699e PMC4410908

[28] Adeleye AS, Keller AA 2014 Long-term colloidal stability and metal leaching of single wall carbon nanotubes: effect of temperature and extracellular polymeric substances. Water Res. 49, 236–250. (doi:10.1016/j.watres.2013.11.032) 24342047 10.1016/j.watres.2013.11.032

[29] George S et al. 2011 Use of a high-throughput screening approach coupled with *in vivo* Zebrafish embryo screening to develop hazard ranking for engineered nanomaterials. ACS Nano 5, 1805–1817. (doi:10.1021/nn102734s) 21323332 10.1021/nn102734s PMC3896549

[30] Torres-Duarte C, Adeleye AS, Pokhrel S, Mädler L, Keller AA, Cherr GN 2016 Developmental effects of two different copper oxide nanomaterials in sea urchin (*Lytechinus pictus*) embryos. Nanotoxicology 10, 671–679. (doi:10.3109/17435390.2015.1107145) 26643145 10.3109/17435390.2015.1107145

[31] Win KY, Feng SS 2005 Effects of particle size and surface coating on cellular uptake of polymeric nanoparticles for oral delivery of anticancer drugs. Biomaterials 26, 2713–2722. (doi:10.1016/j.biomaterials.2004.07.050) 15585275 10.1016/j.biomaterials.2004.07.050

[32] Ji Z et al. 2010 Dispersion and stability optimization of TiO_2_ nanoparticles in cell culture media. Environ. Sci. Technol. 44, 7309–7314. (doi:10.1021/es100417s) 20536146 10.1021/es100417s PMC3971839

[33] Fairbairn EA, Keller AA, Mädler L, Zhou D, Pokhrel S, Cherr GN 2011 Metal oxide nanomaterials in seawater: linking physicochemical characteristics with biological response in sea urchin development. J. Hazard. Mater. 192, 1565–1571. (doi:10.1016/j.jhazmat.2011.06.080) 21775060 10.1016/j.jhazmat.2011.06.080

[34] Keller AA, Wang H, Zhou D, Lenihan HS, Cherr GN, Cardinale BJ, Miller R, Ji Z 2010 Stability and aggregation of metal oxide nanoparticles in natural aqueous matrices. Environ. Sci. Technol. 44, 1962–1967. (doi:10.1021/es902987d) 20151631 10.1021/es902987d

[35] Lynch I, Salvati A, Dawson KA 2009 Protein-nanoparticle interactions: what does the cell see? Nat. Nanotechnol. 4, 546–547. (doi:10.1038/nnano.2009.248) 19734922 10.1038/nnano.2009.248

[36] Walczyk D, Bombelli FB, Monopoli MP, Lynch I, Dawson KA 2010 What the cell ‘sees’ in bionanoscience. J. Am. Chem. Soc. 132, 5761–5768. (doi:10.1021/ja910675v) 20356039 10.1021/ja910675v

[37] Monopoli MP, Walczyk D, Campbell A, Elia G, Lynch I, Bombelli FB, Dawson KA 2011 Physical−chemical aspects of protein corona: relevance to *in vitro* and *in vivo* biological impacts of nanoparticles. J. Am. Chem. Soc. 133, 2525–2534. (doi:10.1021/ja107583h) 21288025 10.1021/ja107583h

[38] Mahmoudi M, Lynch I, Ejtehadi MR, Monopoli MP, Bombelli FB, Laurent S 2011 Protein−nanoparticle interactions: opportunities and challenges. Chem. Rev. 111, 5610–5637. (doi:10.1021/cr100440g) 21688848 10.1021/cr100440g

[39] Lesniak A, Campbell A, Monopoli MP, Lynch I, Salvati A, Dawson KA 2010 Serum heat inactivation affects protein corona composition and nanoparticle uptake. Biomaterials 31, 9511–9518. (doi:10.1016/j.biomaterials.2010.09.049) 21059466 10.1016/j.biomaterials.2010.09.049

[40] Xia T et al. 2011 Decreased dissolution of ZnO by iron doping yields nanoparticles with reduced toxicity in the rodent lung and Zebrafish embryos. ACS Nano 5, 1223–1235. (doi:10.1021/nn1028482) 21250651 10.1021/nn1028482 PMC3900638

[41] Glibowski P, Bukowska A 2011 The effect of pH, temperature and heating time on inulin chemical stability. Acta. Sci. Pol. Technol. Aliment. 10, 189–196.

[42] Adeleye AS, Conway JR, Perez T, Rutten P, Keller AA 2014 Influence of extracellular polymeric substances on the long-term fate, dissolution, and speciation of copper-based nanoparticles. Environ. Sci. Technol. 48, 12 561–12 568. (doi:10.1021/es5033426) 10.1021/es5033426 25295836

[43] Moore M 2006 Do nanoparticles present ecotoxicological risks for the health of the aquatic environment? Environ. Int. 32, 967–976. (doi:10.1016/j.envint.2006.06.014) 16859745 10.1016/j.envint.2006.06.014

