# Supplementary material for: Facilitation of trace metal uptake in cells by inulin coating of metallic nanoparticles
Source: R Soc Open Sci. 2017 Sep 13;4(9):170480. doi: 10.1098/rsos.170480 (PMC5627095; doi:10.1098/rsos.170480)
Supplement: Nanoparticle preparation, zinc uptake assessment, toxicity determination [file rsos170480supp1.docx]

Electronic Supplementary Information for

**Facilitation of trace metal uptake in cells by inulin coating of metallic nanoparticles**

Esmeralda Santillán-Urquiza^1^, Fernando Arteaga-Cardona^2^, Cristina Torres-Duarte^3^, Bryan Cole^4^, Bing Wu^5^, Miguel A. Méndez-Rojas^2^, Gary N. Cherr^3, 6, *^

^a^ Departamento de Ingeniería Química, Ambiental y de Alimentos, Universidad de las Américas Puebla, Puebla, México.

^b^ Departamento de Ciencias Químico-Biológicas, Universidad de las Américas Puebla, Puebla, México.

^c^ Bodega Marine Laboratory, University of California-Davis, Bodega Bay, California, USA.

^d^ School of Veterinary Medicine, University of California-Davis, Davis, California, USA.

^e^ School of the Environment, Nanjing University, Nanjing, PR China.

^f^ Department of Environmental Toxicology and Nutrition, University of California-Davis, Davis, California, USA.

* *Corresponding author:*

Gary N. Cherr

Bodega Marine Laboratory

University of California Davis

PO Box 247

Bodega Bay, CA 94951 USA

E-mail:gncherr@ucdavis.edu

**1. Nanoparticle preparation and characterization**

Zinc (II) chloride (98 %), iron chloride (II) tetrahydrated (99 %), iron (III) chloride hexahydrated (98 %), ammonium hydroxide (25 – 30 % ammonia), sodium hydroxide (97 %) and ethanol (99.93 %) were used. All chemicals were analytical grade from Sigma-Aldrich, Toluca, Mexico, no further purification was done unless otherwise stated and used as received. Inulin (Fructagave SP750, Monterrey, Mexico), a natural polysaccharide with formula C_6n_H_10n+2_O_5n+1_, was used for nanoparticle coating.

*1.1. Preparation of ZnO nanoparticles*

ZnO nanoparticles were synthesized by dissolving ZnCl_2_ in ethanol and then added dropwise to a solution of NaOH in ethanol. The mixture was left stirring for two hours and then washed by centrifugation. The white precipitate was left dried at 30 ºC overnight to obtain a fine white powder.

*1.2. Preparation of α-Fe_2_O_3_@ZnO nanoparticles*

α-Fe_2_O_3_ nanoparticles (n-α-Fe_2_O_3_) were synthetized by fist synthesizing magnetite (Fe_3_O_4_) using a well-known co-precipitation method. The change from the magnetite phase (black powder) to hematite phase (red powder) was carried out by the calcination in air of magnetite in a furnace (Furnace FB1300, Thermolyne, USA) at 500 ºC for 1 hour, to ensure the complete oxidation of magnetite thus change from Fe_3_O_4_ into α-Fe_2_O_3_. The fine red powder obtained was used as seed for the growth of a layer of ZnO. 100 mg of α-Fe_2_O_3_ were added to a 0.10 M solution of ZnCl_2_ in ethanol and heated to 40 ºC for 5 minutes. A solution of NaOH was then dropwise to the mixture and left stirring for 2 hours and then washed twice with ethanol by centrifugation. The powder was then dried overnight at 30 ºC.

*1.3. Inulin coating*

For the inulin coating of the samples, 100 mg of dried nanoparticles were dispersed in 10 mL of distilled water and then 10 mL of a 3% solution of inulin in distilled water was added to the dispersed nanoparticles. The mixture was left stirring for 24 hours. The inulin coated nanoparticles were then washed with distilled water by centrifugation and left at 30 ºC overnight to obtain a dried powder.

*1.4. Characterization*

All samples were characterized by transmission electron microscopy (TEM), dynamic light scattering (DLS) and X-ray powder diffraction. Results are summarized in Table 1 of the main manuscript. Stability of the nanoparticles with and without inulin coating was determined by dispersing 10 mg of each sample in 1 L of distilled water (10 ppm). Zn(II) and Fe(II) concentrations were followed during 7 days and analyzed using atomic absorption spectroscopy (AAS, Varian Spectra AA 220FS, Midland, ON, Canada). Analysis were conducted at different pHs that mimic different scenarios: neutral conditions present in the extracellular media (pH 7), pH 4.5 that simulates the pH found in lysosomes, and pH 2 that simulate the acidic conditions of human digestive tract. Acidification to pH 2 and 4.5 was achieved by adding the appropriate amounts of 6 M HCl or lactic acid, respectively. To determine the amount of dissolution (soluble zinc present in the water), 3 mL aliquots were taken along the 7 days and analyzed by AAS using an air/acetylene flame, with an ionization temperature of 1100°C. Zinc concentrations in the samples was calculated from a calibration curve previously obtained using stock solutions of 20 ppm of ZnCl_2_, (Sigma-Aldrich, atomic spectroscopy standards) digested in HCl 6M at concentrations of 1, 2.5, 5, 7.5 and 10 ppm. All measurements were done in triplicate.

The presence of inulin on the surface of the nanoparticles was determined by FTIR and TGA/DSC measurement.^1^

To determine the amount of soluble zinc present in the media in which hemocytes were exposed, stock solutions (1g/L) of the nanoparticles were first prepared in DI and sonicated for 30 min. Then, 10 mL of a 20ppm solutions (normalized by zinc concentration) were prepared in physiological saline solution (PS) in 6-well polystyrene plate and stored at 14°C. After the indicated time, the nanoparticles were separated from soluble zinc by centrifugation at 14 000×g for 10min in an Eppendorf Microcentrifuge 5417C (Thermo Scientific) using Amicon Ultra-0.5 Ultracel 3 centrifuge tubes (3kDa cutoff, Millipore, Billerica, MA). The concentration of zinc in the eluent was analyzed as described below, and percent of dissolved zinc was calculated based on the total zinc measured in the same sample.

**2. Zinc determination**

A modified version of the method reported by Grand et al. was used for the determination of zinc and has been adapted for a plate reader.^2^ The detection is based in the increase in fluorescence of the FluoZin-3 probe in the presence of soluble zinc. The pH of samples (either seawater or tissue homogenates) and standards was determined before measuring and adjusted when necessary between pH 7 and pH 8. Standards (0-20 ppb final concentration) were freshly prepared everyday using IC TraceCERT®, 1000 mg/L (1 ppt) Zn^2+^ (Sigma-Aldrich, St. Louis, MO). Stock solutions of FluoZin-3 5µM were prepared in ammonium acetate buffer 0.25 M pH 7.5 and stored at -20°C until use.

Samples and standards (125 µL/well) were loaded in triplicate in a black 96-well plate flat bottom. Then, 125 µL/well of a freshly prepared solution of FluoZin-3 200 nM was added with a multichannel pipette. FluoZin-3 200 nM was prepared by diluting the stock solution into the corresponding solvent (PS or filtered seawater). Fluorescence was immediately measured in a TECAN GENios microplate reader (Maennedorf, Switzerland) using an excitation of 485 nm and emission of 530 nm. Concentration of the sample was determined using the standard curve obtained from the same plate. An example of a standard curve is shown in Supplemental Figure S1. To validate this method, samples of different concentrations were prepared in filtered seawater and measured using this method and at the University of California Santa Barbara using Inductively Coupled Plasma Atomic Emission Spectroscopy (ICP-AES). Values are presented in Supplemental Table S1.


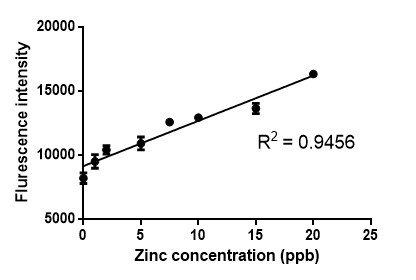


**Supplemental Fig. S1** Zinc standard curve in filtered seawater measured by fluorescence.

Supplemental Table S1. Comparison of zinc concentrations determined by ICP-AES and by fluorescence in a plate reader.

| Zinc concentration determined by ICP-AES (ppb) | Zinc concentration determined by fluorescence (ppb) |
| --- | --- |
| 180 | 157 |
| 206 | 199 |
| 373 | 351 |
| 1560 | 1371 |

**3. Cellular responses of hemocytes exposed to carbon nanotubes, silver and copper oxide nanoparticles**

Hemocytes were exposed to different ENMs whose toxicity has been previously demonstrated in other model systems.^3-6^ Copper oxide nanoparticles (nCuO) were obtained from Sigma-Aldrich (St. Louis, MO), and has been previously characterized.^7^ Single wall carbon nanotubes (CNT) and nanosized silver spheres (nAg, 20 nm diameter) were provided by the University of California's Center for Environmental Implications of Nanotechnology (Los Angeles, CA), and their properties have been previously reported.^4,7^ Nanoparticle suspensions and exposures were conducted following the same protocol described in Materials and Methods sections 2.2-2.4. The cellular responses varied for each nanomaterial (Supplemental Fig. S2). Exposure to nAg caused a decrease in cell viability as well as an increase in lysosome abundance only at 10 ppm. nCuO affected both cell viability and cell function at concentrations of 5 ppm and higher. CNT was the only nanomaterial that caused an increase in reactive oxygen species production when present at 20 ppm, and increased the lysosome abundance when present at 10 ppm and 20 ppm.


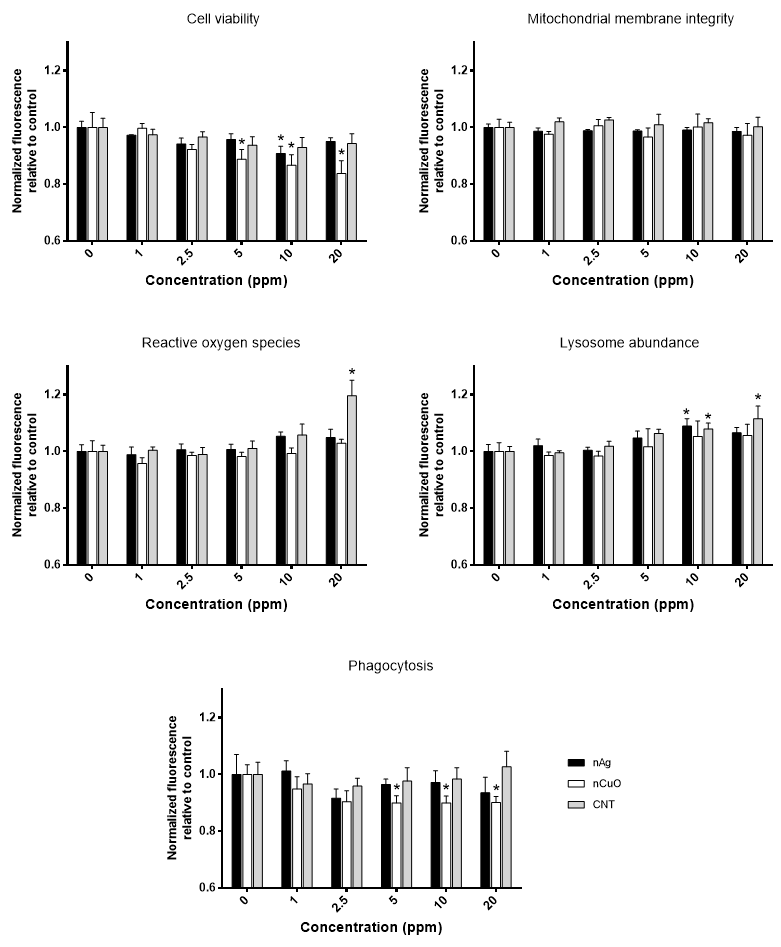


**Supplemental Fig. S2** Cellular responses of mussel hemocytes exposed to silver nanoparticles (nAg), copper oxide nanoparticles (nCuO) and single wall carbon nanotubes(CNT). The asterisk indicates statistically significant differences (p˂0.05) compared to the 0 ppm (control) treatment of the corresponding chemical treatment. Values are average ± standard error (n≥4).

**References.**

1. E. Santillán-Urquiza, F. Arteaga-Cardona, E. Hernandez-Herman, P. Pacheco-García, R. González-Rodríguez, J. L. Coffer, M. Mendoza-Alvarez, J. F. Vélez-Ruiz, M. A. Méndez-Rojas, *J. Colloid Interface Sci.*, 2015, **460**, 339-348.
2. M. Grand, H. M. Oliveira, J. Ruzicka, C. Measures, *Analyst*, 2011, **136**, 2747-2755.
3. A. S. Adeleye, J. R. Conway, T. Perez, P. Rutten, A. A. Keller, *Environ. Sci. Technol.*, 2014, **48**, 12561-12568.
4. S. George, S. Lin, Z. Ji, C. R. Thomas, L. Li, M. Mecklenburg, H. Meng, X. Wang, H. Zhang, T. Xia, J. N. Hohman, J. I. Zink, P. S. Weiss, A. E. Nel, *ACS Nano*, 2012, **6**, 3745-3759.
5. C. Torres-Duarte, A. S. Adeleye, S. Pokhrel, L. Mädler, A. A. Keller, G. N. Cherr, *Nanotoxicology*, 2016, **10**, 671-679.
6. B. Wu, C. Torres-Duarte, B. Cole, G. N. Cherr, *Environ. Sci. Technol.*, 2015, **49**, 5760-5770.
7. A. S. Adeleye, A. A. Keller, *Water Res.* 2014, **49**, 236-250.

Data

**Cellular responses analyzed in plate reader**

Fluorescence intensity of the corresponding probe normalized by the fluorescence intensity of Hoechst 33342.

**Cell death (EtHD1)**

| Replicate | Zinc concentration from ZnSO_4_ (ppm) | | | | | |
| --- | --- | --- | --- | --- | --- | --- |
|  | 0 | 1 | 2.5 | 5 | 10 | 20 |
| 1 | 1.85 | 1.81 | 1.80 | 1.79 | 1.80 | 1.87 |
| 2 | 1.81 | 1.80 | 1.80 | 1.79 | 1.83 | 1.88 |
| 3 | 1.65 | 1.60 | 1.59 | 1.57 | 1.63 | 1.55 |
| 4 | 1.50 | 1.49 | 1.45 | 1.44 | 1.47 | 1.55 |

| Replicate | Inulin concentration (ppm) | | | | | |
| --- | --- | --- | --- | --- | --- | --- |
|  | 0 | 1 | 2.5 | 5 | 10 | 20 |
| 1 | 1.89 | 1.81 | 1.80 | 1.77 | 1.81 | 1.81 |
| 2 | 1.88 | 1.81 | 1.67 | 1.81 | 1.85 | 1.90 |
| 3 | 1.84 | 1.83 | 1.82 | 1.82 | 1.84 | 1.90 |
| 4 | 1.55 | 1.51 | 1.53 | 1.51 | 1.55 | 1.60 |

| Replicate | Zinc concentration from nZnO (ppm) | | | | | |
| --- | --- | --- | --- | --- | --- | --- |
|  | 0 | 1 | 2.5 | 5 | 10 | 20 |
| 1 | 1.87 | 1.79 | 1.77 | 1.80 | 1.81 | 1.70 |
| 2 | 1.82 | 1.84 | 1.69 | 1.82 | 1.78 | 1.86 |
| 3 | 2.13 | 2.25 | 2.28 | 2.31 | 2.26 | 2.13 |
| 4 | 1.61 | 1.55 | 1.54 | 1.54 | 1.56 | 1.63 |
| 5 | 1.19 | 1.19 | 1.19 | 1.18 | 1.18 | 1.36 |

| Replicate | Zinc concentration from nZnO inulin (ppm) | | | | | |
| --- | --- | --- | --- | --- | --- | --- |
|  | 0 | 1 | 2.5 | 5 | 10 | 20 |
| 1 | 1.82 | 1.78 | 1.78 | 1.79 | 1.79 | 1.87 |
| 2 | 1.88 | 1.83 | 1.84 | 1.86 | 1.89 | 1.93 |
| 3 | 1.66 | 1.58 | 1.58 | 1.52 | 1.55 | 1.64 |
| 4 | 1.49 | 1.46 | 1.49 | 1.52 | 1.50 | 1.55 |

| Replicate | Zinc concentration from nα-Fe2O3@ZnO (ppm) | | | | | |
| --- | --- | --- | --- | --- | --- | --- |
|  | 0 | 1 | 2.5 | 5 | 10 | 20 |
| 1 | 1.83 | 1.80 | 1.78 | 1.79 | 1.83 | 1.83 |
| 2 | 1.82 | 1.81 | 1.83 | 1.78 | 1.83 | 1.86 |
| 3 | 1.63 | 1.58 | 1.60 | 1.57 | 1.60 | 1.65 |
| 4 | 1.14 | 0.90 | 1.01 | 1.14 | 1.06 | 1.29 |

| Replicate | Zinc concentration from nα-Fe2O3@ZnO inulin (ppm) | | | | | |
| --- | --- | --- | --- | --- | --- | --- |
|  | 0 | 1 | 2.5 | 5 | 10 | 20 |
| 1 | 1.84 | 1.85 | 1.83 | 1.81 | 1.82 | 1.87 |
| 2 | 1.86 | 1.84 | 1.85 | 1.82 | 1.85 | 1.90 |
| 3 | 1.57 | 1.49 | 1.48 | 1.50 | 1.51 | 1.55 |
| 4 | 1.21 | 1.12 | 1.21 | 1.19 | 1.33 | 1.43 |

**Cell viability (CAM)**

| Replicate | Zinc concentration from ZnSO_4_ (ppm) | | | | | |
| --- | --- | --- | --- | --- | --- | --- |
|  | 0 | 1 | 2.5 | 5 | 10 | 20 |
| 1 | 0.41 | 0.44 | 0.45 | 0.43 | 0.42 | 0.43 |
| 2 | 0.39 | 0.39 | 0.39 | 0.39 | 0.40 | 0.41 |
| 3 | 0.49 | 0.50 | 0.52 | 0.46 | 0.48 | 0.41 |
| 4 | 0.62 | 0.60 | 0.64 | 0.62 | 0.56 | 0.58 |

| Replicate | Inulin concentration (ppm) | | | | | |
| --- | --- | --- | --- | --- | --- | --- |
|  | 0 | 1 | 2.5 | 5 | 10 | 20 |
| 1 | 0.43 | 0.46 | 0.44 | 0.43 | 0.42 | 0.41 |
| 2 | 0.41 | 0.40 | 0.40 | 0.40 | 0.41 | 0.43 |
| 3 | 0.41 | 0.39 | 0.39 | 0.39 | 0.40 | 0.41 |
| 4 | 0.46 | 0.42 | 0.43 | 0.43 | 0.43 | 0.44 |

| Replicate | Zinc concentration from nZnO (ppm) | | | | | |
| --- | --- | --- | --- | --- | --- | --- |
|  | 0 | 1 | 2.5 | 5 | 10 | 20 |
| 1 | 0.41 | 0.41 | 0.43 | 0.42 | 0.41 | 0.43 |
| 2 | 0.45 | 0.53 | 0.56 | 0.57 | 0.60 | 0.75 |
| 3 | 0.43 | 0.87 | 0.57 | 0.66 | 0.53 | 0.55 |
| 4 | 0.55 | 0.59 | 0.56 | 0.55 | 0.52 | 0.49 |

| Replicate | Zinc concentration from nZnO inulin (ppm) | | | | | |
| --- | --- | --- | --- | --- | --- | --- |
|  | 0 | 1 | 2.5 | 5 | 10 | 20 |
| 1 | 0.46 | 0.52 | 0.46 | 0.48 | 0.47 | 0.53 |
| 2 | 0.41 | 0.40 | 0.42 | 0.43 | 0.45 | 0.46 |
| 3 | 0.44 | 0.46 | 0.48 | 0.50 | 0.45 | 0.46 |
| 4 | 0.26 | 0.25 | 0.50 | 0.51 | 0.40 | 0.32 |

| Replicate | Zinc concentration from nα-Fe2O3@ZnO (ppm) | | | | | |
| --- | --- | --- | --- | --- | --- | --- |
|  | 0 | 1 | 2.5 | 5 | 10 | 20 |
| 1 | 0.42 | 0.43 | 0.42 | 0.41 | 0.42 | 0.43 |
| 2 | 0.39 | 0.41 | 0.43 | 0.41 | 0.41 | 0.44 |
| 3 | 0.46 | 0.46 | 0.50 | 0.55 | 0.49 | 0.46 |
| 4 | 1.49 | 1.05 | 1.03 | 0.98 | 1.01 | 1.05 |

| Replicate | Zinc concentration from nα-Fe2O3@ZnO inulin (ppm) | | | | | |
| --- | --- | --- | --- | --- | --- | --- |
|  | 0 | 1 | 2.5 | 5 | 10 | 20 |
| 1 | 1.20 | 1.01 | 0.86 | 0.77 | 0.70 | 0.70 |
| 2 | 0.42 | 0.42 | 0.43 | 0.43 | 0.42 | 0.44 |
| 3 | 0.52 | 0.59 | 0.57 | 0.61 | 0.54 | 0.52 |
| 4 | 1.17 | 1.03 | 1.37 | 1.34 | 1.32 | 1.40 |

**Lysosome abundance (Lysotracker)**

| Replicate | Zinc concentration from ZnSO_4_ (ppm) | | | | | |
| --- | --- | --- | --- | --- | --- | --- |
|  | 0 | 1 | 2.5 | 5 | 10 | 20 |
| 1 | 2.74 | 2.54 | 2.72 | 2.65 | 2.80 | 2.95 |
| 2 | 2.76 | 2.63 | 2.58 | 2.68 | 2.77 | 2.93 |
| 3 | 2.57 | 2.49 | 2.53 | 2.61 | 2.55 | 2.66 |
| 4 | 2.42 | 2.47 | 2.45 | 2.33 | 2.54 | 2.57 |

| Replicate | Inulin concentration (ppm) | | | | | |
| --- | --- | --- | --- | --- | --- | --- |
|  | 0 | 1 | 2.5 | 5 | 10 | 20 |
| 1 | 2.73 | 2.68 | 2.72 | 2.60 | 2.79 | 2.81 |
| 2 | 2.84 | 2.75 | 2.77 | 2.76 | 2.90 | 2.95 |
| 3 | 2.98 | 2.90 | 2.79 | 2.90 | 2.94 | 3.00 |
| 4 | 2.58 | 2.50 | 2.47 | 2.44 | 2.56 | 2.54 |

| Replicate | Zinc concentration from nZnO (ppm) | | | | | |
| --- | --- | --- | --- | --- | --- | --- |
|  | 0 | 1 | 2.5 | 5 | 10 | 20 |
| 1 | 2.78 | 2.88 | 2.61 | 2.71 | 2.77 | 2.88 |
| 2 | 2.95 | 2.83 | 2.80 | 2.84 | 2.92 | 3.01 |
| 3 | 2.79 | 2.30 | 2.29 | 2.31 | 2.26 | 2.49 |
| 4 | 2.66 | 2.50 | 2.42 | 2.44 | 2.58 | 2.62 |
| 5 | 2.06 | 2.06 | 2.07 | 2.01 | 2.28 | 2.25 |

| Replicate | Zinc concentration from nZnO inulin (ppm) | | | | | |
| --- | --- | --- | --- | --- | --- | --- |
|  | 0 | 1 | 2.5 | 5 | 10 | 20 |
| 1 | 2.94 | 2.85 | 2.86 | 2.60 | 2.90 | 2.89 |
| 2 | 2.91 | 2.88 | 2.85 | 2.86 | 2.91 | 2.95 |
| 3 | 2.58 | 2.53 | 2.48 | 2.49 | 2.51 | 2.56 |
| 4 | 2.64 | 2.48 | 2.51 | 2.49 | 2.50 | 2.51 |

| Replicate | Zinc concentration from nα-Fe2O3@ZnO (ppm) | | | | | |
| --- | --- | --- | --- | --- | --- | --- |
|  | 0 | 1 | 2.5 | 5 | 10 | 20 |
| 1 | 2.92 | 2.74 | 2.82 | 2.80 | 2.80 | 2.99 |
| 2 | 2.89 | 2.93 | 2.82 | 2.81 | 2.96 | 3.02 |
| 3 | 2.56 | 2.50 | 2.51 | 2.54 | 2.48 | 2.69 |
| 4 | 2.07 | 2.22 | 2.13 | 2.43 | 2.29 | 2.51 |

| Replicate | Zinc concentration from nα-Fe2O3@ZnO inulin (ppm) | | | | | |
| --- | --- | --- | --- | --- | --- | --- |
|  | 0 | 1 | 2.5 | 5 | 10 | 20 |
| 1 | 2.92 | 2.73 | 2.76 | 2.77 | 2.75 | 2.92 |
| 2 | 3.04 | 2.85 | 2.91 | 2.96 | 2.96 | 3.10 |
| 3 | 2.68 | 2.59 | 2.53 | 2.50 | 2.56 | 2.65 |
| 4 | 2.30 | 2.22 | 2.26 | 2.18 | 2.30 | 2.43 |

**Mitochondria membrane potential (JC1)**

| Replicate | Zinc concentration from ZnSO_4_ (ppm) | | | | | |
| --- | --- | --- | --- | --- | --- | --- |
|  | 0 | 1 | 2.5 | 5 | 10 | 20 |
| 1 | 4.13 | 3.90 | 3.97 | 3.87 | 3.89 | 3.80 |
| 2 | 3.82 | 3.54 | 3.34 | 3.37 | 3.50 | 3.52 |
| 3 | 4.54 | 4.45 | 4.32 | 4.38 | 4.37 | 4.39 |
| 4 | 4.85 | 4.84 | 4.41 | 4.70 | 4.66 | 4.58 |

| Replicate | Inulin concentration (ppm) | | | | | |
| --- | --- | --- | --- | --- | --- | --- |
|  | 0 | 1 | 2.5 | 5 | 10 | 20 |
| 1 | 4.28 | 3.92 | 3.96 | 3.87 | 3.89 | 3.72 |
| 2 | 3.96 | 3.85 | 4.00 | 3.78 | 3.74 | 3.64 |
| 3 | 3.67 | 3.85 | 4.09 | 4.05 | 4.15 | 4.01 |
| 4 | 5.06 | 4.60 | 4.70 | 4.60 | 4.55 | 4.76 |

| Replicate | Zinc concentration from nZnO (ppm) | | | | | |
| --- | --- | --- | --- | --- | --- | --- |
|  | 0 | 1 | 2.5 | 5 | 10 | 20 |
| 1 | 5.00 | 4.57 | 4.46 | 4.23 | 4.13 | 4.10 |
| 2 | 3.76 | 3.77 | 3.42 | 3.57 | 3.42 | 3.92 |
| 3 | 4.32 | 3.46 | 3.53 | 3.48 | 3.50 | 3.43 |
| 4 | 4.23 | 4.28 | 4.43 | 4.46 | 4.59 | 4.55 |
| 5 | 4.90 | 4.84 | 5.43 | 4.91 | 5.07 | 5.10 |

| Replicate | Zinc concentration from nZnO inulin (ppm) | | | | | |
| --- | --- | --- | --- | --- | --- | --- |
|  | 0 | 1 | 2.5 | 5 | 10 | 20 |
| 1 | 4.45 | 4.11 | 3.86 | 3.98 | 4.04 | 4.19 |
| 2 | 5.10 | 4.40 | 4.74 | 4.98 | 5.27 | 5.33 |
| 3 | 4.72 | 4.42 | 4.32 | 4.31 | 4.33 | 4.41 |
| 4 | 19.95 | 11.57 | 10.04 | 6.75 | 6.77 | 9.89 |

| Replicate | Zinc concentration from nα-Fe2O3@ZnO (ppm) | | | | | |
| --- | --- | --- | --- | --- | --- | --- |
|  | 0 | 1 | 2.5 | 5 | 10 | 20 |
| 1 | 4.03 | 3.61 | 3.32 | 3.75 | 3.75 | 3.62 |
| 2 | 4.13 | 3.78 | 3.80 | 3.97 | 3.71 | 4.00 |
| 3 | 5.88 | 5.22 | 5.32 | 4.06 | 4.94 | 5.27 |
| 4 | 4.96 | 4.84 | 5.27 | 5.26 | 5.32 | 5.32 |

| Replicate | Zinc concentration from nα-Fe2O3@ZnO inulin (ppm) | | | | | |
| --- | --- | --- | --- | --- | --- | --- |
|  | 0 | 1 | 2.5 | 5 | 10 | 20 |
| 1 | 3.84 | 3.71 | 3.58 | 4.41 | 3.95 | 3.99 |
| 2 | 3.41 | 3.54 | 3.61 | 3.62 | 4.32 | 4.66 |
| 3 | 5.36 | 4.66 | 5.03 | 5.12 | 4.78 | 4.99 |
| 4 | 6.28 | 5.49 | 5.47 | 5.32 | 5.32 | 4.99 |

**Reactive oxygen species (DCF)**

| Replicate | Zinc concentration from ZnSO_4_ (ppm) | | | | | |
| --- | --- | --- | --- | --- | --- | --- |
|  | 0 | 1 | 2.5 | 5 | 10 | 20 |
| 1 | 0.37 | 0.38 | 0.35 | 0.37 | 0.37 | 0.38 |
| 2 | 0.37 | 0.38 | 0.37 | 0.38 | 0.39 | 0.41 |
| 3 | 0.43 | 0.38 | 0.38 | 0.38 | 0.39 | 0.40 |
| 4 | 0.42 | 0.41 | 0.40 | 0.38 | 0.37 | 0.39 |

| Replicate | Inulin concentration (ppm) | | | | | |
| --- | --- | --- | --- | --- | --- | --- |
|  | 0 | 1 | 2.5 | 5 | 10 | 20 |
| 1 | 0.37 | 0.37 | 0.37 | 0.37 | 0.38 | 0.38 |
| 2 | 0.41 | 0.41 | 0.41 | 0.42 | 0.40 | 0.41 |
| 3 | 0.40 | 0.40 | 0.38 | 0.39 | 0.39 | 0.39 |
| 4 | 0.43 | 0.38 | 0.39 | 0.34 | 0.37 | 0.41 |

| Replicate | Zinc concentration from nZnO (ppm) | | | | | |
| --- | --- | --- | --- | --- | --- | --- |
|  | 0 | 1 | 2.5 | 5 | 10 | 20 |
| 1 | 0.43 | 0.41 | 0.42 | 0.42 | 0.43 | 0.45 |
| 2 | 0.42 | 0.42 | 0.43 | 0.42 | 0.42 | 0.43 |
| 3 | 0.41 | 0.40 | 0.41 | 0.38 | 0.36 | 0.39 |
| 4 | 0.44 | 0.41 | 0.41 | 0.42 | 0.42 | 0.43 |
| 5 | 0.47 | 0.38 | 0.42 | 0.36 | 0.43 | 0.41 |

| Replicate | Zinc concentration from nZnO inulin (ppm) | | | | | |
| --- | --- | --- | --- | --- | --- | --- |
|  | 0 | 1 | 2.5 | 5 | 10 | 20 |
| 1 | 0.42 | 0.42 | 0.42 | 0.40 | 0.42 | 0.41 |
| 2 | 0.38 | 0.39 | 0.37 | 0.38 | 0.38 | 0.39 |
| 3 | 0.47 | 0.44 | 0.41 | 0.42 | 0.41 | 0.41 |
| 4 | 0.27 | 0.24 | 0.28 | 0.32 | 0.27 | 0.24 |

| Replicate | Zinc concentration from nα-Fe2O3@ZnO (ppm) | | | | | |
| --- | --- | --- | --- | --- | --- | --- |
|  | 0 | 1 | 2.5 | 5 | 10 | 20 |
| 1 | 0.38 | 0.40 | 0.39 | 0.40 | 0.40 | 0.39 |
| 2 | 0.39 | 0.39 | 0.39 | 0.39 | 0.39 | 0.38 |
| 3 | 0.43 | 0.43 | 0.40 | 0.41 | 0.45 | 0.38 |
| 4 | 0.35 | 0.42 | 0.41 | 0.36 | 0.36 | 0.42 |

| Replicate | Zinc concentration from nα-Fe2O3@ZnO inulin (ppm) | | | | | |
| --- | --- | --- | --- | --- | --- | --- |
|  | 0 | 1 | 2.5 | 5 | 10 | 20 |
| 1 | 0.44 | 0.42 | 0.40 | 0.40 | 0.40 | 0.39 |
| 2 | 0.42 | 0.41 | 0.40 | 0.40 | 0.39 | 0.39 |
| 3 | 0.43 | 0.44 | 0.45 | 0.43 | 0.41 | 0.40 |
| 4 | 0.42 | 0.38 | 0.45 | 0.38 | 0.38 | 0.44 |

**Intracellular zinc concentration (NPG)**

| Replicate | Zinc concentration from ZnSO_4_ (ppm) | | | | | |
| --- | --- | --- | --- | --- | --- | --- |
|  | 0 | 1 | 2.5 | 5 | 10 | 20 |
| 1 | 0.38 | 0.39 | 0.40 | 0.41 | 0.40 | 0.41 |
| 2 | 0.39 | 0.40 | 0.33 | 0.40 | 0.42 | 0.42 |
| 3 | 0.46 | 0.42 | 0.44 | 0.46 | 0.54 | 0.50 |
| 4 | 0.44 | 0.47 | 0.47 | 0.44 | 0.46 | 0.54 |

| Replicate | Inulin concentration (ppm) | | | | | |
| --- | --- | --- | --- | --- | --- | --- |
|  | 0 | 1 | 2.5 | 5 | 10 | 20 |
| 1 | 0.36 | 0.36 | 0.37 | 0.36 | 0.37 | 0.36 |
| 2 | 0.37 | 0.38 | 0.39 | 0.39 | 0.39 | 0.40 |
| 3 | 0.44 | 0.43 | 0.42 | 0.42 | 0.43 | 0.43 |
| 4 | 0.36 | 0.35 | 0.32 | 0.32 | 0.32 | 0.33 |

| Replicate | Zinc concentration from nZnO (ppm) | | | | | |
| --- | --- | --- | --- | --- | --- | --- |
|  | 0 | 1 | 2.5 | 5 | 10 | 20 |
| 1 | 0.38 | 0.41 | 0.41 | 0.42 | 0.44 | 0.43 |
| 2 | 0.42 | 0.42 | 0.43 | 0.43 | 0.43 | 0.47 |
| 3 | 0.38 | 0.33 | 0.34 | 0.34 | 0.36 | 0.49 |
| 4 | 0.43 | 0.43 | 0.45 | 0.46 | 0.47 | 0.53 |
| 5 | 0.30 | 0.32 | 0.32 | 0.32 | 0.36 | 0.36 |

| Replicate | Zinc concentration from nZnO inulin (ppm) | | | | | |
| --- | --- | --- | --- | --- | --- | --- |
|  | 0 | 1 | 2.5 | 5 | 10 | 20 |
| 1 | 0.42 | 0.44 | 0.44 | 0.44 | 0.71 | 0.70 |
| 2 | 0.40 | 0.41 | 0.46 | 0.52 | 0.53 | 0.59 |
| 3 | 0.48 | 0.50 | 0.50 | 0.53 | 0.59 | 0.89 |
| 4 | 0.43 | 0.45 | 0.47 | 0.50 | 0.61 | 0.72 |

| Replicate | Zinc concentration from nα-Fe2O3@ZnO (ppm) | | | | | |
| --- | --- | --- | --- | --- | --- | --- |
|  | 0 | 1 | 2.5 | 5 | 10 | 20 |
| 1 | 0.41 | 0.42 | 0.41 | 0.42 | 0.42 | 0.43 |
| 2 | 0.43 | 0.42 | 0.44 | 0.44 | 0.43 | 0.44 |
| 3 | 0.46 | 0.46 | 0.44 | 0.44 | 0.47 | 0.47 |
| 4 | 0.31 | 0.33 | 0.35 | 0.39 | 0.38 | 0.39 |

| Replicate | Zinc concentration from nα-Fe2O3@ZnO inulin (ppm) | | | | | |
| --- | --- | --- | --- | --- | --- | --- |
|  | 0 | 1 | 2.5 | 5 | 10 | 20 |
| 1 | 0.45 | 0.43 | 0.45 | 0.47 | 0.52 | 0.58 |
| 2 | 0.43 | 0.43 | 0.43 | 0.43 | 0.45 | 0.48 |
| 3 | 0.46 | 0.51 | 0.54 | 0.57 | 0.56 | 0.76 |
| 4 | 0.34 | 0.36 | 0.37 | 0.38 | 0.39 | 0.52 |

**Intracellular zinc concentrations**

Zinc concentrations measured in the 2 mL samples where 800,000 hemocytes were homogenized by sonication. To determine the concentration per cell in pg/hemocyte, multiply by 2.375x10^-3^. Negative values were considered zero.

| Treatment | Zinc concentration (ppb) | | | |
| --- | --- | --- | --- | --- |
|  | 1 | 2 | 3 | 4 |
| Control | -7 | -1 | -3 | -4 |
| ZnSO_4_ | 9 | 11 | 17 | 19 |
| nZnO | 27 | 10 | 23 | 39 |
| nZnO inulin | 2051 | 1333 | 1625 | 1721 |
| nα-Fe2O3@ZnO | 98 | 106 | 55 | 54 |
| nα-Fe2O3@ZnO inulin | 1234 | 1324 | 1186 | 1154 |
| Inulin | 18 | 20 |  |  |
| nZnO + inulin | 72 | 64 |  |  |
| ZnSO_4_ + inulin | 15 | 13 |  |  |

**Phagocytosis**

Each treatment was replicated in 3 wells (identified as 1, 2 and 3). For each well, 3 images were taken (identified as A, B and C). The number of phagocytic and non-phagocytic cells was later analyzed. Results are shown below.

| Treatment | Phagocytosed? | Well | | | | | | | | |
| --- | --- | --- | --- | --- | --- | --- | --- | --- | --- | --- |
|  |  | 1 | | | 2 | | | 3 | | |
|  |  | A | B | C | A | B | C | A | B | C |
| Control | Yes | 63 | 71 | 52 | 47 | 44 | 50 | 30 | 42 | 36 |
|  | No | 57 | 41 | 33 | 28 | 29 | 27 | 34 | 32 | 27 |
| ZnSO_4_ | Yes | 29 | 63 | 28 | 42 | 27 | 49 | 28 | 33 | 26 |
|  | No | 41 | 40 | 33 | 40 | 47 | 57 | 30 | 38 | 39 |
| nZnO | Yes | 43 | 45 | 38 | 33 | 49 | 35 | 28 | 30 | 26 |
|  | No | 61 | 64 | 68 | 79 | 47 | 50 | 47 | 37 | 28 |
| nZnO nulin | Yes | 20 | 25 | 12 | 14 | 11 | 21 | 33 | 30 | 17 |
|  | No | 47 | 43 | 39 | 28 | 43 | 32 | 54 | 32 | 43 |
| nα-Fe2O3@ZnO | Yes | 25 | 29 | 35 | 23 | 36 | 29 | 20 | 32 |  |
|  | No | 49 | 52 | 55 | 47 | 53 | 52 | 35 | 40 |  |
| nα-Fe2O3@ZnO inulin | Yes | 39 | 27 | 37 | 15 | 25 | 26 | 33 | 16 | 24 |
|  | No | 56 | 54 | 58 | 39 | 48 | 39 | 43 | 54 | 46 |
| Inulin | Yes | 64 | 28 | 46 | 22 | 49 | 39 | 26 | 35 |  |
|  | No | 56 | 47 | 56 | 34 | 55 | 53 | 29 | 32 |  |
| nZnO + Inulin | Yes | 28 | 37 | 29 | 28 | 28 | 25 | 40 | 38 | 30 |
|  | No | 33 | 45 | 46 | 52 | 39 | 45 | 46 | 59 | 50 |
